# *Shewanella* spp. Bloodstream Infections in Queensland, Australia

**DOI:** 10.3201/eid2804.212193

**Published:** 2022-04

**Authors:** Kevin B. Laupland, Adam G. Stewart, Felicity Edwards, David L. Paterson, Sonali Coulter, Claire Heney, Narelle George, Patrick Harris

**Affiliations:** Royal Brisbane and Women’s Hospital, Brisbane, Queensland, Australia (K.B. Laupland, A.G. Stewart, D.L. Paterson);; Queensland University of Technology, Brisbane (K.B. Laupland, F. Edwards);; University of Queensland, Brisbane (A.G. Stewart, D.L. Paterson, P. Harris);; Medication Services Queensland, Brisbane (S. Coulter);; Pathology Queensland, Brisbane (C. Heney, N. George, P. Harris)

**Keywords:** Shewanella spp., bacteria, bloodstream infections, BSIs, marine bacteria, antimicrobial resistance, Australia

## Abstract

The epidemiology of bloodstream infections caused by *Shewanella* spp. is not well defined. Our objective was to define the incidence and determinants of *Shewanella* spp. bloodstream infections by using population-based surveillance in Queensland, Australia during 2000‒2019. The incidence was 1.0 cases/1 million persons annually and was highest during summer and in the tropical Torres and Cape region. Older persons and male patients were at highest risk. At least 1 concurrent condition was documented in 75% of case-patients, and 30-day all cause case-fatality rate was 15%. Aging populations in warm climates might expect an increasing burden of these infections.

## Abstract 2

Aging populations in warm climates might expect an increasing burden of these infections.

*Shewanella* spp., most commonly *S. algae* and *S. putrefaciens*, are infrequent but occasionally severe causes of human infection associated with exposure to warm marine environments (*1*,*2*). Cases of otogenic and skin and soft tissue infections caused by *S.* (*Pseudomonas*) *putrefaciens* were described in the 1960s, and case series of bacteremic infections were reported in subsequent decades (*3*,*4*). Vignier et al. reported 16 cases of *Shewanella* spp. infection that occurred in Martinique during 1997–2012 and identified an additional 239 cases in review of the published literature during 1973–2011 (*5*). That study observed that otogenic, skin and soft tissue, abdominal/biliary tract, and respiratory tract foci of infection were most common, and that 71 (28%) cases were bacteremic (*5*). *Shewanella* spp. are frequently coisolated with other organisms, most notably Enterobacterales and *Aeromonas* and *Vibrio* spp., and occasionally might develop major antimicrobial drug resistance (*2*,*5*,*6*).

As a result of their rarity, the epidemiology of *Shewanella* spp. bloodstream infections (BSIs) is poorly defined. The existing body of literature is limited to case reports and small series. In addition, as a result of developments in genomic and phenotypic testing, it has been recognized that before the current millennium many reports of *S. putrefaciens* infection might have been actually caused by *S. algae* (*7*). The objective of this study was to determine the contemporary incidence of and risk factors for development of *Shewanella* spp. BSIs in the population of Queensland, Australia.

## Methods

The study population was all residents (2019 population ≈5 million) of Queensland, Australia. Queensland is a large state with diverse geography that includes subtropical and tropical coastal regions and inland dry desert areas. Approximately two thirds of the population is concentrated around the Greater Brisbane/Gold Coast/Sunshine Coast areas in the southeastern corner of the state, and the remainder is distributed predominantly along the eastern coastal areas. Healthcare within the publicly funded system is administered through 16 hospital and healthcare service regions (*8*). All Queensland residents with incident BSIs caused by *Shewanella* spp. identified by Pathology Queensland during January 1, 2000‒December 31, 2019, were included in this study. Approval of the health research ethics committee at the Royal Brisbane and Women’s Hospital was granted with a waiver of individual consent (LNR/2020/QRBW/62494).

During the study, we used different methods to identify *Shewanella* spp.. Before 2008, VITEK 1, API20E, API20NE, (all from bioMérieux), and Microscan (Baxter Diagnostics Inc., https://www.baxter.com) were used to identify *Shewanella* spp., but these methods were not able to identify *S. algae*. From ≈2008 onward, the VITEK database for gram-negative identification was upgraded to improve differentiation of *S. algae*. After matrix-assisted laser desorption/ionization time-of-flight mass spectrometry was introduced during 2012, differentiation of the 2 *Shewanella* spp. was possible. In some instances in which earlier methods could not reliably distinguish between *S. putrefaciens* and *S. algae*, growth in nutrient broth with and without 6% NaCl was used to confirm the species identification. In instances in which methods were inadequate to reliably differentiate species, isolates are reported as *Shewanella* spp.. We performed antimicrobial drug susceptibility testing by using an automated method (i.e., VITEK AST Card, bioMérieux) and disc diffusion according to recognized standards (Clinical and Laboratory Standards Institute, https://clsi.org) at the time of testing.

All blood cultures that had growth of *Shewanella* spp. were retrospectively identified by the Clinical Information Systems Support Unit, Queensland Health, during the study period. We defined incident BSIs by the first isolation of a *Shewanella* spp. for a patient; all subsequent isolations of the same species from that patient within 30 days were deemed to represent the same episode. Polymicrobial infections were those from which a *Shewanella* spp. was coisolated with >1 other major pathogens within a 48-hour period. Two independent sets of cultures were required for common contaminants to define significance (*9*).

Once incident episodes were identified, we obtained additional clinical and outcome information by using linkages to statewide databases. We identified all healthcare encounters with private and public institutions within the 2 years before 1 year after index blood cultured within the Queensland Hospital Admitted Patient Data Collection. We used this collection to determine healthcare encounters and hospital admission and discharge dates, discharge survival status, as well as all diagnostic codes (International Classification of Diseases, 10th Revision, Australian modification). Multiple admission episodes occurring within a continuous time period (such as with interhospital transfers) were deemed to represent 1 hospital admission for purposes of length of stay. We queried the Registry of General Deaths (https://info.australia.gov.au) as of December 31, 2020, to identify all deaths.

We classified BSIs as hospital-onset if the index blood culture was obtained 2 calendar days after admission or within 2 calendar days of hospital discharge (*10*). BSIs infections diagnosed in the community or within the first 2 calendar days of stay in hospital were classified as community-onset. Healthcare-associated BSIs were those community-onset BSIs that fulfilled >1 criteria: nursing home resident, encounter at a healthcare institution within 30 days, or admission to a hospital for >2 days within 90 days before index blood culture (*10*). We classified community-onset BSIs that did not fulfill criteria for healthcare-associated infections as community-associated. We defined comorbid medical illnesses by using Charlson comorbidity index and established these illnesses by using validated coding dictionaries (*11*,*12*). We assigned a clinical focus on the basis of review of diagnosis-related group and primary hospital discharge codes.

We analyzed data by using Stata version 16.1 (StataCorp LLC, https://www.stata.com). The primary unit of analysis was incident BSI episodes, reported as crude incident rates per million persons annually. We excluded cases identified among persons who were not Queensland residents and obtained denominator data stratified by age, sex, and hospital and health service area from Queensland Health (*13*). We obtained the total annual number of sets of blood cultures performed by Pathology Queensland to calculate the overall culturing rate per population as described (*14*). We obtained average monthly mean peak and low temperatures and rainfall from Queensland weather stations from the Australian Bureau of Meteorology (*15*). Incidence rate differences were expressed as incidence rate ratios and reported with exact 95% CIs. p values <0.05 represented statistical significance.

## Results

During >86 million person-years of surveillance, 86 *Shewanella* spp. BSIs occurred to give an incidence of 1.0 cases/1 million Queensland residents/year. We identified 4 additional episodes of *Shewanella* spp. BSIs for persons residing in other states in Australia, and we excluded them from analysis. No second episodes of incident *Shewanella* spp. BSIs occurred. Most (65, 76%) cases of BSIs were caused by *S.* *algae*, 4 (5%) were caused by *S. putrefaciens*, and 17 (20%) were not identified to the species level.

**Figure 1:**
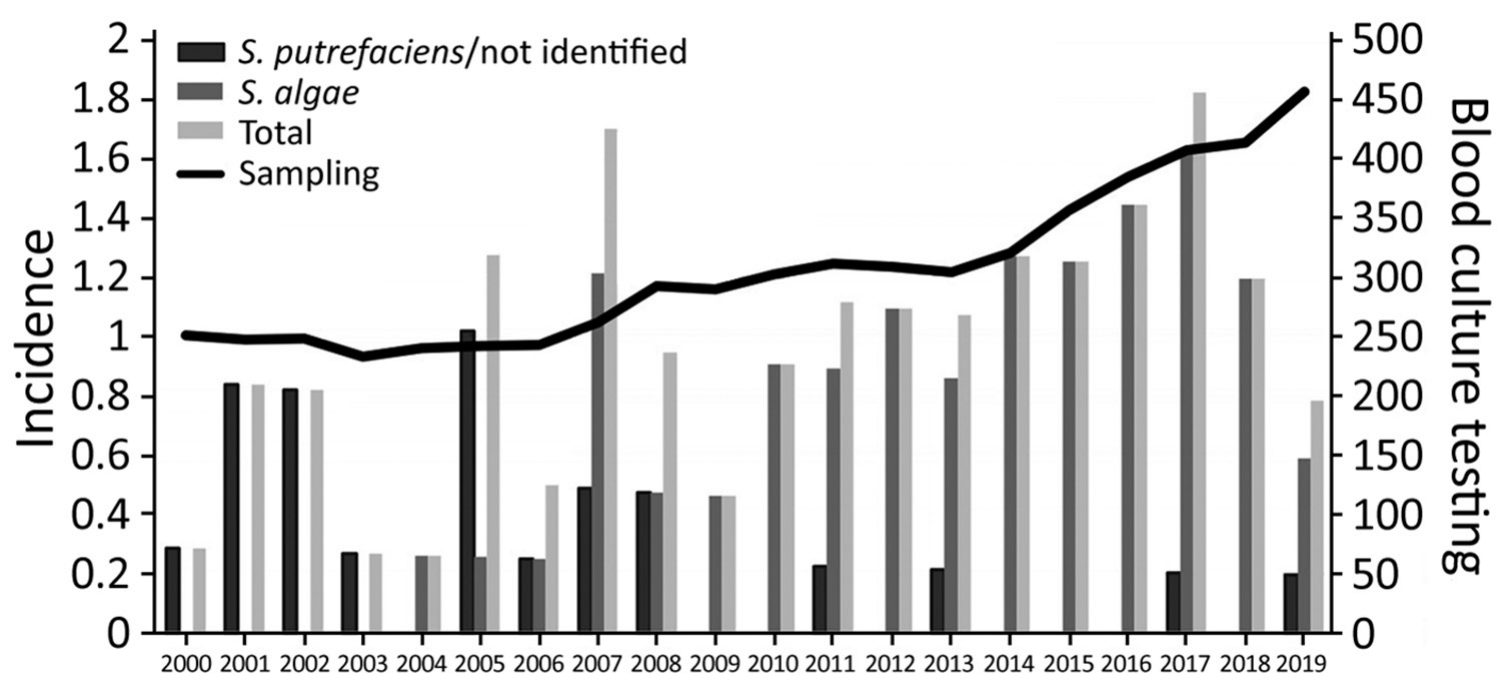
Incidence (cases/1 million persons) of *Shewanella* species bloodstream infections and number of blood samples collected per 1 million persons, Queensland, Australia.

**Figure 2:**
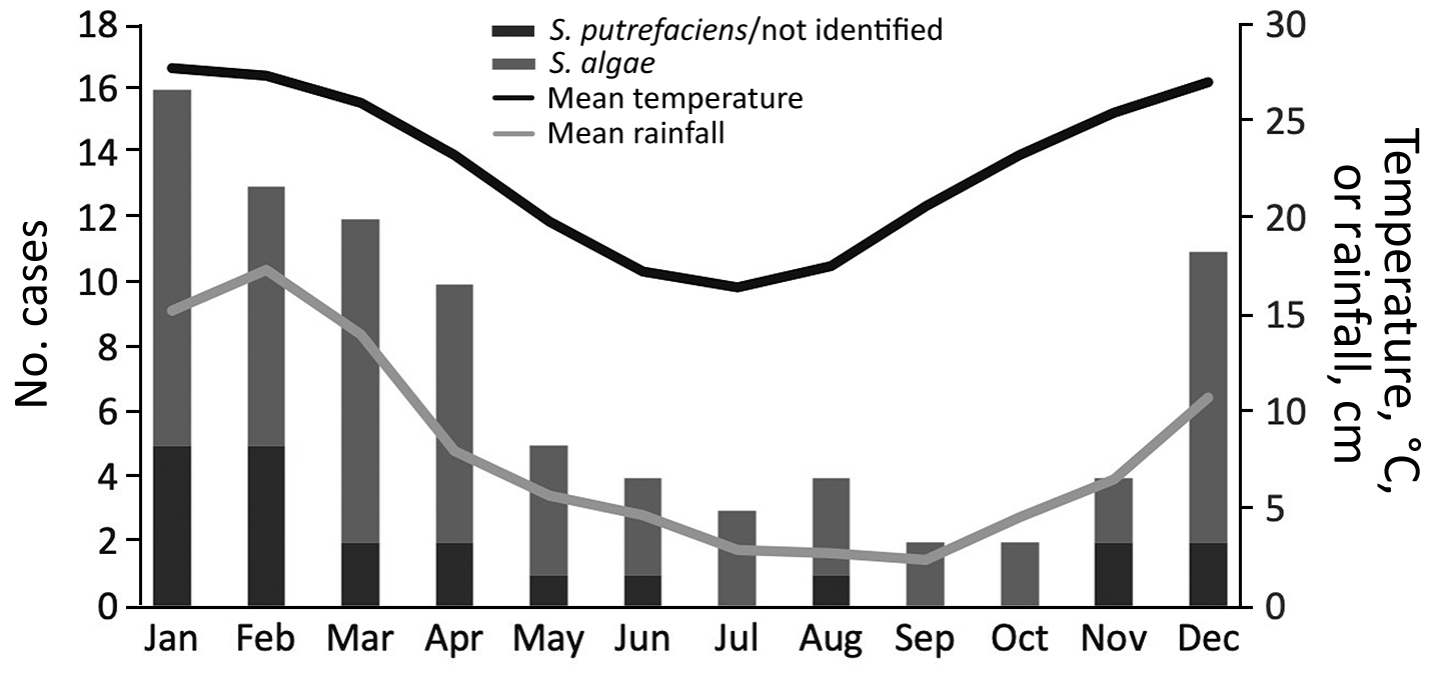
Monthly occurrence of *Shewanella* species bloodstream infections and mean temperature and rainfall, Queensland, Australia.

There was moderate year-to-year variability in the overall incidence of *Shewanella* spp. BSIs (Figure 1). Occurrence of cases of *Shewanella* spp. BSIs varied according to the month of the year; there was a peak in the warmer, wetter months and nadir in the drier, cooler months (Figure 2).

**Figure 3:**
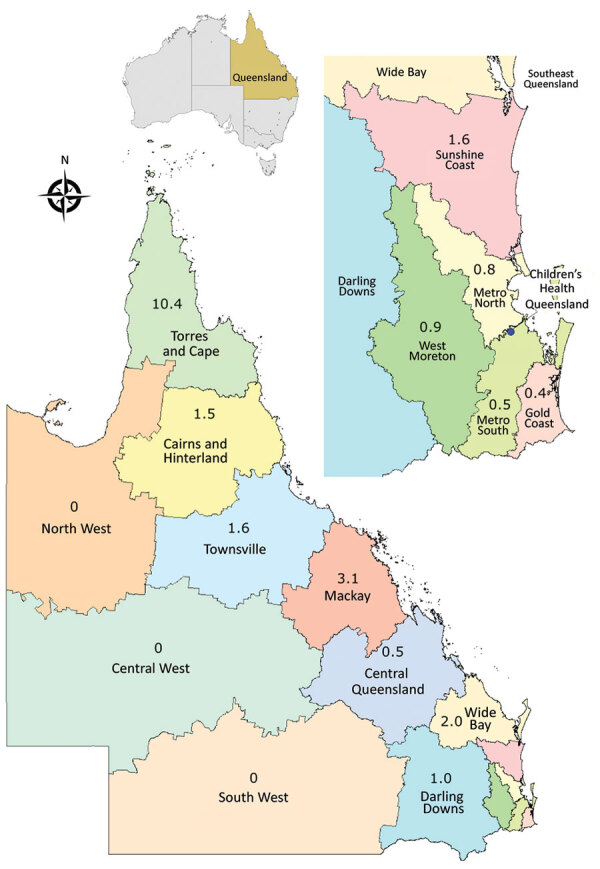
Incidence of *Shewanella* species bloodstream infections (cases/1 million persons), by hospital and health service, Queensland, Australia. Inset at top right shows enlarged coastal area; inset at top shows location of Queensland in Australia. Numbers indicate incidence. Adapted with permission from Queensland Health, https://www.health.qld.gov.au.

Incidence of *Shewanella* spp. BSIs varied considerably among the regions of the state (Figure 3). No cases were observed within the western outback regions, and low rates were observed in the Greater Brisbane area. The highest rates occurred in the tropical coastal Torres and Cape area (Figure 3).

**Figure 4:**
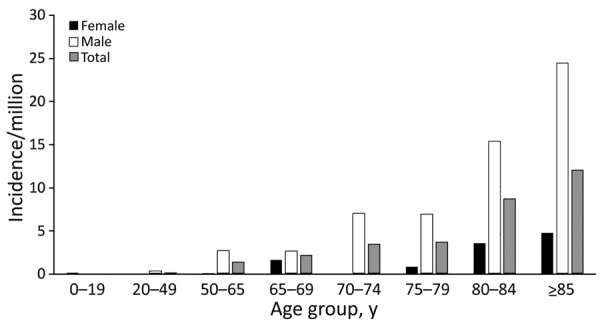
Age-specific and sex-specific incidence (cases/1 million persons) of *Shewanella* species bloodstream infections, Queensland, Australia.

The median age of case-patients was 71.4 (interquartile range [IQR] 60.3–82.8) years, and 72 (84%) incident episodes were in male patients. There was an increased risk for development of *Shewanella* species BSIs with advancing age, particularly among male patients (Figure 4). Male patients had an overall 5-fold increased risk compared with female patients (1.7 cases/1 million vs. 0.3 cases/1 million; incidence rate ratios for male patients 5.2 [95% CI 2.9–9.0]; p<0.0001).

Most BSIs were community-onset; 25 (29%) were classified as healthcare-associated, 54 (64%) as community-associated, and 6 (7%) as hospital-onset. Of the 25 healthcare-associated case-patients, 21 (84%) had hospital visits within 30 days, and 10 (40%) had hospital admissions within 90 days before the index episode. None were nursing home residents. The median Charlson comorbidity index was 2 (IQR 1–3), and only 21 (25%) patients had no underlying conditions documented. The most common focus of infection was soft tissue (35 cases, 41%) cases. Seven (8%) case-patients had an abdominal focus, 6 (7%) had a lower respiratory focus, 2 (2%) had an endovascular focus, and 1 case each had a bone/joint, head/neck, or genitourinary focus of infection. No focus was identified for 33 (38%) case-patients.

Most (73, 70%) infections were monomicrobial. Among the 13 polymicrobial infections, 4 patients had 3 isolates, and 2 patients had 4 isolates. The co-isolated organisms included *Escherichia coli* in 7 cases, and 1 each of *Candida* spp., *Corynebacterium diphtheriae*, *Enterobacter cloacae*, *Klebsiella oxytoca*, *Proteus mirabilis*, *Sphingomonas paucimobilis*, *Pseudomonas oryzihabitans*, *Pseudomonas aeruginosa*, *Stenotrophomonas maltophilia*, group G streptococcus, *Vibrio vulnificus*, and 1 unidentified gram-negative bacillus. Four isolates (n = 65; 6%) were meropenem resistant and 1 (n = 70; 1%) was ceftazidime resistant. No resistance to gentamicin (n = 76) or ciprofloxacin (n = 74) was observed among *Shewanella* spp. isolates tested.

All but 1 patient was admitted to a hospital for management (median length of stay 8 [IQR 5–16] days). Four patients required admission to an intensive care unit. Twelve (14%) patients died during the index hospitalization, and 13 (15%) died within 30 days of BSI diagnosis. Among the 13 patients who died, 6 (46%) had no focus identified, 3 (23%) had an abdominal focus, and 2 (15%) had soft tissue and lower respiratory tract infections. Eleven (85%) patients who died had >1 Charlson comorbidity.

## Discussion

We describe the epidemiology of *Shewanella* species BSIs in a large population in Australia. We confirm these infections as rare infections and provide novel incidence data. In addition, although usually considered community-associated pathogens, 29% of our cases were healthcare-associated and 7% hospital-onset. We also observed that the most patients had underlying medical illnesses and that the elderly were at highest risk. This finding is useful given that that the Queensland population, like those in many other high-income countries, is aging and showing an increased prevalence of chronic illnesses. Therefore, we might expect that the burden of *Shewanella* species BSIs will increase in the coming years.

There are few previous contemporary studies for which to compare our results. Although case series have been published since the turn of the millennium, they have included small numbers of cases, of which only a minority have been associated with BSIs (*2*,*5*). Our observation of a temporal relationship between seasonal temperature and rainfall is much more pronounced than when similarly examined in a previous study conducted in Reunion Island (*2*). Climate factors probably play a major role in these infections given that they are usually identified in warm regions. However, these infections probably involve a complex interaction between environmental and human activity‒related factors.

Vignier et al. reported a case series from Martinique and summarized the world literature on *Shewanella* species infections during 2013 (*5*). Although that study provided useful results, similar to all summaries of case reports, these results must be interpreted with caution because unusual or atypical cases might be more likely to be submitted and accepted for publication. Therefore, summaries of such reports might not reflect the average or usual characteristics of such infections. Our study has the benefit of comprehensive and consistent identification of all cases identified by a statewide laboratory such that selection biases are minimized and secular changes over time might be observed.

Nearly all of the *Shewanella* species BSIs occurring in our population during years when we had adequate means of identification were caused by *S. algae*. It has been recognized that many *S. putrifaciens* cases previously reported were probably *S. algae* because the clinical characteristics of these infections might be confused (*5*,*7*). Unfortunately, we do not have all of our original isolates for retesting.

Our study benefited from surveillance of a large population in Queensland. Including BSI data from public hospital and community collection sites over a 20-year period resulted in >86 million person-years of observations. The relatively small number of BSIs attributed to *Shewanella* species (86) limited the statistical significance of further analysis of the data, such as differences between species, underlying conditions, and outcome. A strength of our study is that our laboratory surveillance was statewide in scope and included specimens sent from hospital and community collection sites.

However, our study was limited to the publicly funded system. Thus, cases identified within private hospitals and collection sites were not included. Although we speculate that this represents a small proportion of missed cases, our incidence rates should be interpreted as potential underestimates of the true population rate. A second limitation was that our study was retrospective; therefore, we were restricted in data variables for analysis. It would have been informative to examine environmental exposures, such as risk factors and the effect of specific antimicrobial drug treatments on outcome (*16*). A third limitation was that as a result of small numbers, we did not age and sex standardize our incidence rates. The possibility exists that at least some of the increased incidence observed in recent years of the study could have been related to demographic changes. Finally, as is the case for any study examining BSIs, our ability to detect a case is dependent on whether clinicians obtain blood for culturing from patients who have suspected infections. In addition, only a portion of all disease attributable to *Shewanella* species is associated with positive blood cultures. Accordingly, the true rate of disease attributable to *Shewanella* species in our population is likely higher than we detected (*14*).

In conclusion, we present a novel study of *Shewanella* species BSIs that details the epidemiology of these infections in a large population in Australia. We observe that older persons are at highest risk and that their incidence is increased in association with higher environmental temperatures. Although *Shewanella* species BSIs are rare, there is a major potential for large increases in coming years as a result of aging populations and climate change. Ongoing surveillance is warranted.
